# Effect of Silibinin in Reducing Inflammatory Pathways in In Vitro and In Vivo Models of Infection-Induced Preterm Birth

**DOI:** 10.1371/journal.pone.0092505

**Published:** 2014-03-19

**Authors:** Ratana Lim, Carrington J. Morwood, Gillian Barker, Martha Lappas

**Affiliations:** 1 Mercy Perinatal Research Centre, Mercy Hospital for Women, Heidelberg, Victoria, Australia; 2 Obstetrics, Nutrition and Endocrinology Group, Department of Obstetrics and Gynaecology, University of Melbourne, Victoria, Australia; University of Leuven, Rega Institute, Belgium

## Abstract

Infection-induced preterm birth is the largest cause of infant death and of neurological disabilities in survivors. Silibinin, from milk thistle, exerts potent anti-inflammatory activities in non-gestational tissues. The aims of this study were to determine the effect of silibinin on pro-inflammatory mediators in (i) human fetal membranes and myometrium treated with bacterial endotoxin lipopolysaccharide (LPS) or the pro-inflammatory cytokine IL-1β, and (ii) in preterm fetal membranes with active infection. The effect of silibinin on infection induced inflammation and brain injury in pregnant mice was also assessed. Fetal membranes and myometrium (tissue explants and primary cells) were treated with 200 μM silibinin in the presence or absence of 10 μg/ml LPS or 1 ng/ml IL-1β. C57BL/6 mice were injected with 70 mg/kg silibinin with or without 50 μg LPS on embryonic day 16. Fetal brains were collected after 6 h. In human fetal membranes, silibinin significantly decreased LPS-stimulated expression of IL-6 and IL-8, COX-2, and prostaglandins PGE_2_ and PGF_2α_. In primary amnion and myometrial cells, silibinin also decreased IL-1β-induced MMP-9 expression. Preterm fetal membranes with active infection treated with silibinin showed a decrease in IL-6, IL-8 and MMP-9 expression. Fetal brains from mice treated with silibinin showed a significant decrease in LPS-induced IL-8 and ninjurin, a marker of brain injury. Our study demonstrates that silibinin can reduce infection and inflammation-induced pro-labour mediators in human fetal membranes and myometrium. Excitingly, the *in vivo* results indicate a protective effect of silibinin on infection-induced brain injury in a mouse model of preterm birth.

## Introduction

Preterm birth is the single leading cause of neonatal death worldwide, after exclusion of congenital defects, and can lead to numerous long-term health consequences for those surviving babies [1]. Spontaneous preterm birth, which accounts for almost 70% of all preterm births, may result from preterm labour with intact membranes or preterm pre-labour rupture of membranes (PPROM) [2]. Intrauterine infection, commonly presented as chorioamnionitis, is an acute inflammation of the membranes and chorion of the placenta, typically due to ascending bacterial infection. Chorioamnionitis complicates a third of patients with preterm labour [3] and is the most common complication associated with PPROM [4]. Chorioamnionitis predisposes the preterm infant to numerous organ disease, affecting cardiopulmonary, cerebral, and renal systems [5]; developmental delay and lifelong neurological impairments, such as mental retardation, cerebral palsy and learning and behavioural deficits, are caused by perinatal brain damage [6]–[8]. Besides the emotional burden on families, direct and indirect costs of preterm birth accounts for billions of health care dollars each year.

Labour, as an inflammatory event, is associated with increased expression of pro-inflammatory mediators in intrauterine tissues. These include pro-inflammatory cytokines IL-6, IL-8, TNF-α and IL-1β in the cervix, myometrium, fetal membranes and placenta [9]-[12]; prostaglandins PGE_2_ and PGF_2α_ in the cervix, fetal membranes and myometrium [13]; and matrix metalloproteinase (MMP)-9 in the fetal membranes and placenta [14]. The regulation of these mediators leads to tissue remodelling of the cervix and fetal membranes, myometrial contractions, and membrane rupture, leading to successful delivery at term. It is untimely activation of these processes that can lead to spontaneous preterm birth [2].

There is no single drug that can treat all the underlying mechanisms that lead to spontaneous preterm birth. While there are currently measures to delay preterm labour, in an effort to provide enough time to administer antenatal corticosteroids or to transport the mother to a tertiary care facility, each have limitations and possible side effects. Tocolytics can delay preterm labour for a short time, including magnesium sulphate, beta-mimetics, calcium channel blockers, prostaglandin inhibitors and oxytocin receptor antagnonists [15]; however, the use of tocolytics is controversial due to the lack of evidence of significant benefits in neonatal outcomes [16]. Progesterone treatment has been associated with a reduction in rates of preterm birth and risk of infant birthweight of less than 2500 g [17]. Limitations include maternal side effects and a lack of information regarding optimal doses, mode of administration and gestation to commence therapy (prior to, or after 20 weeks gestation), as well as longer-term infant and childhood outcomes. Some clinical trials in progress include the tocolytic nifedipine [18], [19], nifedipine based on cervical length [20], and dietary supplementation of the omega-3 long-chain polyunsaturated fatty acid docosahexaenoic acid (DHA), which resulted in longer gestation and larger infant size [21].

Silibinin, also known as silybin, is the main active compound of silymarin, the standardised extract of milk thistle. Silibinin has a long history of safe use by humans, exhibiting extremely low toxicity [22]. Silibinin has been utilised as an effective treatment for hepatic disease [23] and as an adjunctive cancer therapy [24], [25]. Silibinin has been shown to have potent anti-inflammatory and anti-cancer effects (reviewed in [26]); silibinin reduced pro-inflammatory mediators *in vitro* in canine hepatocytes [27], an HIV viral infection model [28], *in vitro* and *in vivo* models of mouse skin inflammation [29], [30], and skin and prostate cancer cells from animal models [24]. In mouse models of allergic inflammation [31] and infection-induced sepsis [32], silibinin decreased inflammation and increased survival rate. Chelation of iron may be a mode of action in the anti-cancer properties of silibinin [33]. While the effect of silibinin on reactive oxygen species and pro-inflammatory cytokines has been explored in peripheral blood monocytes from women with preeclampsia [34], [35], there are no other studies describing the effect of silibinin on labour mediators in human gestational tissues. Thus, the aims of this study were to determine the effect of silibinin on pro-inflammatory mediators in (i) human fetal membranes and myometrium treated with bacterial endotoxin lipopolysaccharide (LPS) or the pro-inflammatory cytokine IL-1β, and (ii) in preterm fetal membranes with chorioamnionitis. Further, the *in vivo* effect of silibinin was determined in a mouse model, where LPS was used to induce inflammation in C57BL/6 time mated mice. Maternal tissues and fetal brains were assessed for markers of inflammation and brain injury.

## Materials and Methods

### Ethics

Written informed consent was obtained from all participating patients. Ethics approval was obtained from the Mercy Hospital for Women's Research and Ethics Committee. Pregnant women were recruited to the study by a clinical research midwife.

### Tissue collection

Human placentae with attached fetal membranes and myometrium were collected for two studies: from women who delivered at (i) term (>37 weeks gestation) at elective Caesarean section (indications for Caesarean section were breech presentation and/or previous Caesarean section) in the absence of labour; and (ii) preterm (<37 weeks gestation) with or without chorioamniontis. All tissues were obtained within 15 min of delivery.

For the term studies, fetal membranes, obtained 2 cm from the periplacental edge, and myometrial biopsies, obtained from the upper margin of the incision made in the lower uterine segment, were obtained from women who delivered healthy, singleton infants from elective Caesarean section in the absence of labour. Indications for Caesarean section included repeat Caesarean section or breech presentation. Women with any underlying medical conditions such as diabetes, asthma, polycystic ovarian syndrome, preeclampsia and macrovascular complications were excluded. Additionally, women with multiple pregnancies, obese women, fetuses with chromosomal abnormalities were excluded.

For the preterm studies, fetal membranes were obtained 2 cm from the periplacental edge (n = 7 patients). All the preterm placentas were swabbed for microbiological culture investigations and assessed for histopathological evidence of infection. Chorioamnionitis was diagnosed pathologically according to standard criteria which included histological evidence of macrophages and neutrophils permeating the chorionic cell layer and often infiltrating the amniotic cell. Four of the cases had histologically proven chorioamnionitis from mild to severe. The chorioamnionitis tissues were collected from women either after spontaneous labour onset at Caesarean section or after spontaneous vaginal delivery. The preterm women without infection all went into spontaneous labour and delivered vaginally or at Caesarean section (n = 3 patients). The mean gestation was 30±1.9 weeks; five of the women had PPROM with rupture occurring from 2 to 26 days before delivery (two cases with PPROM were negative for chorioamnionitis). None of the women had any underlying medical conditions such as diabetes, asthma, polycystic ovarian syndrome, preeclampsia and macrovascular complications. Additionally, women with multiple pregnancies, obese women, fetuses with chromosomal abnormalities were excluded.

### Tissue explants

For the term studies, fetal membranes (combined amnion and choriodecidua) and myometrium (collected as described above) were dissected and tissue fragments placed in DMEM at 37°C in a humidified atmosphere of 8% O_2_ and 5% CO_2_ for 1 h. Tissues were blotted dry on sterile filter paper and transferred to 24-well tissue culture plates (100 mg wet weight/well for fetal membranes and 50 mg wet weight/well for myometrium). The explants were incubated, in duplicate, in 1 ml DMEM containing penicillin G (100 U/ml) and streptomycin (100 μg/ml). Explants were pre-incubated with 200 μM silibinin (S0417, Sigma, St. Louis, USA) for 1 h, then incubated, for 24 h, in the presence of 10 μg/ml LPS (to facilitate the production of pro-inflammatory mediators) [36]–[38]. After 24 h incubation, tissue and media were collected separately and stored at −80°C for further analysis as detailed below. Experiments were performed in fetal membranes and myometrium from six patients. Based on previously published studies [39]–[41], a dose response was initially performed to determine the optimal concentration of silibinin. Fetal membranes were incubated in the absence or presence of 10 μg/ml LPS and silibinin at 50, 100 and 200 μM. While all concentrations of silibinin decreased LPS-stimulated IL-6 release, treatment with 200 μM silibinin was closer to basal readings, and was thus used in subsequent experiments (data not shown).

For the preterm study, the effect of silibinin was determined in fetal membranes that either already had an infection or had undergone preterm labour. Explants were incubated with or without 200 μM silibinin for 24 h. After 24 h incubation, tissue and media were collected separately and stored at −80°C for further analysis as detailed below. Experiments were performed from seven patients. For the preterm studies, due to the large variability in basal release or expression of the endpoints, all data were normalised to the untreated samples (basal), which was set at 1.

### Primary amnion and myometrial cell culture

Primary amnion epithelial cultures and myometrium cells were used to investigate the effects of silibinin on pro-labour mediators. The amnion and myometrial cells were stimulated with IL-1β, as LPS does not induce an inflammatory response (observed in our laboratory, also by [42]). For these studies, reflected amnion (obtained approximately 2 cm from the peri-placental edge) was obtained from women who delivered healthy, singleton infants at term (>37 weeks gestation) undergoing elective Caesarean section in the absence of labour. Amnion cells were prepared as previously described [37], [38], [43]–[47]. Myometrial cells were isolated and cultured as previously described [37], [38], [48], [49]. Primary cells at 80–90% confluence were incubated in 1 ng/ml IL-1β (amnion) or 200 pg/ml IL-1β (myometrium) in the absence or presence of 200 μM silibinin. After 24 h incubation, cells and media were collected separately and stored at -80°C for further analysis as detailed below. The medium was assayed for cytokine and prostaglandin release, and cells were assayed for cytokine, COX-2 and MMP-9 gene expression. Experiments were performed on cells collected from six patients.

### Model of infection-induced inflammation in the pregnant mouse

Animal studies were conducted with approval from the Austin Health's Animal Ethics Committee (A2012/04451). Time mated C57BL/6 mice were obtained from the University of Adelaide on day 12 of gestation and allowed to acclimatise for 4 days prior to experiments. They received food and water *ad libitum* and were on a 12-hour light/dark cycle. An initial dose response was performed to determine the optimal dose to induce inflammation in gestational tissues and fetal brain. Based on other studies [50]–[52], 10–75 μg of LPS per mouse was administered intraperitoneally, with the 50 μg/mouse dose being the final concentration used. The concentration of silibinin used was 70 mg/kg, based on other studies [53]–[55] as well as solubility of our reagent. For these studies, on day 16, the mice were intraperitoneally injected with 500 μl of either saline (n = 7), LPS (n = 6), or LPS with silibinin (n = 5). As silibinin was solubilised in DMSO, equal amounts of DMSO (10% v/v) were included in the injections for control and LPS groups. Six hours after injection, mice were euthanised with carbon dioxide and cervical dislocation. This time point was based on the LPS dose response where inflammation was induced, but before preterm delivery occured. Myometrium and placenta were harvested, as well as fetal brains from the pups. Tissues were flash frozen then stored at −80°C until further analysis by qRT-PCR as detailed below.

### Cytokine and prostaglandin assays

Conditioned medium from cell and tissue culture experiments was assessed for IL-6 and IL-8 concentrations using commercial ELISA according to the manufacturer's instructions (Invitrogen, Carlsbad, USA). The concentration of PGE_2_ and PGF_2α_ into the incubation medium were assayed using commercially available competitive enzyme immunoassay kits according to the manufacturer's specifications (Kookaburra Kits from Sapphire Bioscience, Redfern, Australia). The calculated interassay and intraassay coefficients of variation (CV) were all less than 10%. Data was corrected for total protein and expressed as either ng or pg per mg protein. The protein content of tissue homogenates was determined using BCA protein assay (Pierce, Rockford, USA), using BSA as a reference standard, as previously described [56]. For the preterm explant studies, due to patient variability, data were normalised to the untreated samples (basal), which was set at 1.

### Gelatin zymography

Assessment of enzymes of ECM weakening and rupture (MMP-9) was performed by gelatin zymography as previously described [37], [38], [44], [46], [47] on conditioned media collected from primary amnion and myometrium cells, and myometrial explants. Proteolytic activity was visualised as clear zones of lysis on a blue background of undigested gelatin. For the term explant studies, data were corrected for background, and fold change was calculated relative to LPS or IL-1β-stimulated cells, which was set at 1. For the preterm explant studies, data were normalised to the untreated samples (basal), which was set at 1.

### RNA extraction and qRT-PCR

Analysis of human gene expression by qRT-PCR was performed as we have previously described [37], [38], [46]-[49]. Total RNA from cells and tissues was extracted using TRIsure according to manufacturer's instructions (Bioline, London, UK). RNA concentrations were quantified using a spectrophotometer (NanoDrop, Thermo Fisher Scientific, Waltham, USA). RNA quality and integrity was determined via the A260/A280 ratio. One μg of RNA was converted to cDNA using the Tetro cDNA Synthesis Kit (Bioline) according to the manufacturer's instructions. The cDNA was diluted ten-fold and 4 μl of this was used to perform qRT-PCR using SensiFAST SYBR No-ROX kit (Bioline) and 100 nM of primers: GAPDH (QT01192646), IL-6 (QT00083720), IL-8 (QT00000322), COX-2 (QT00040586), MMP-9 (QT00040040) (Qiagen, Germantown, Maryland, USA). The specificity of the product was assessed from melting curve analysis. RNA without reverse transcriptase during cDNA synthesis as well as PCR reactions using water instead of template showed no amplification. Average gene C_T_ values were normalised to the average GAPDH mRNA C_T_ values of the same cDNA sample. For the term explant studies, fold differences in target gene expression were determined by the comparative C_T_ method, relative to LPS or IL-1β treatment. For the myometrial cells, as we have previously published [49], due to variability of patient response to IL-1β, data was expressed as fold change relative to IL-1β-stimulated cells, which was set at 1. For the preterm explant studies, data were normalised to the untreated samples (basal), which was set at 1.

For the mouse studies, RNA was extracted as described above. Mouse primers used for qRT-PCR were GAPDH [57], IL-6 [58], IL-8 [59], IL-1β [60], COX-2 [61] and ninjurin [62]. For the mouse studies, data were normalised to the control mice, which was set at 1.

### Statistical analysis

Statistical analyses were performed using a commercially available statistical software package (Statgraphics Plus version 3.1, Statistical Graphics Corp., Rockville, USA). Homogeneity of data was assessed by Bartlett's test, and when significant, data were logarithmically transformed before further analysis. Data were analysed by one-way ANOVA, and comparisons between groups (to LPS or IL-1β treatment for human studies, and LPS for mice studies) were performed using LSD multiple-range tests. Statistical difference was indicated by a *P* value of less than 0.05. Data are expressed as mean ± standard error of the mean (SEM).

## Results

### Effect of silibinin on pro-inflammatory cytokines in human gestational tissues

The first aim of this study was to determine the effect of silibinin on infection (LPS) and inflammation (IL-1β) induced pro-labour mediators in human fetal membranes and myometrium. Human fetal membranes (combined amnion and choriodecidua) and myometrium were incubated with 200 μM silibinin in the absence or presence of LPS for 24 h. Primary amnion cells and primary myometrial cells were incubated with 200 μM silibinin in the absence or presence of IL-1β for 24 h. The concentration of silibinin was determined from an initial dose response using 50, 100 and 200 μM of silibinin in the presence of LPS. All concentrations of silibinin decreased LPS-stimulated IL-6 concentration, however treatment with 200 μM give a similar reading to basal (data not shown) and was thus used in subsequent experiments. IL-6 and IL-8 gene expression was quantified by qRT-PCR and the concentration of IL-6 and IL-8 in conditioned media assessed by ELISA.

**Figure 1:**
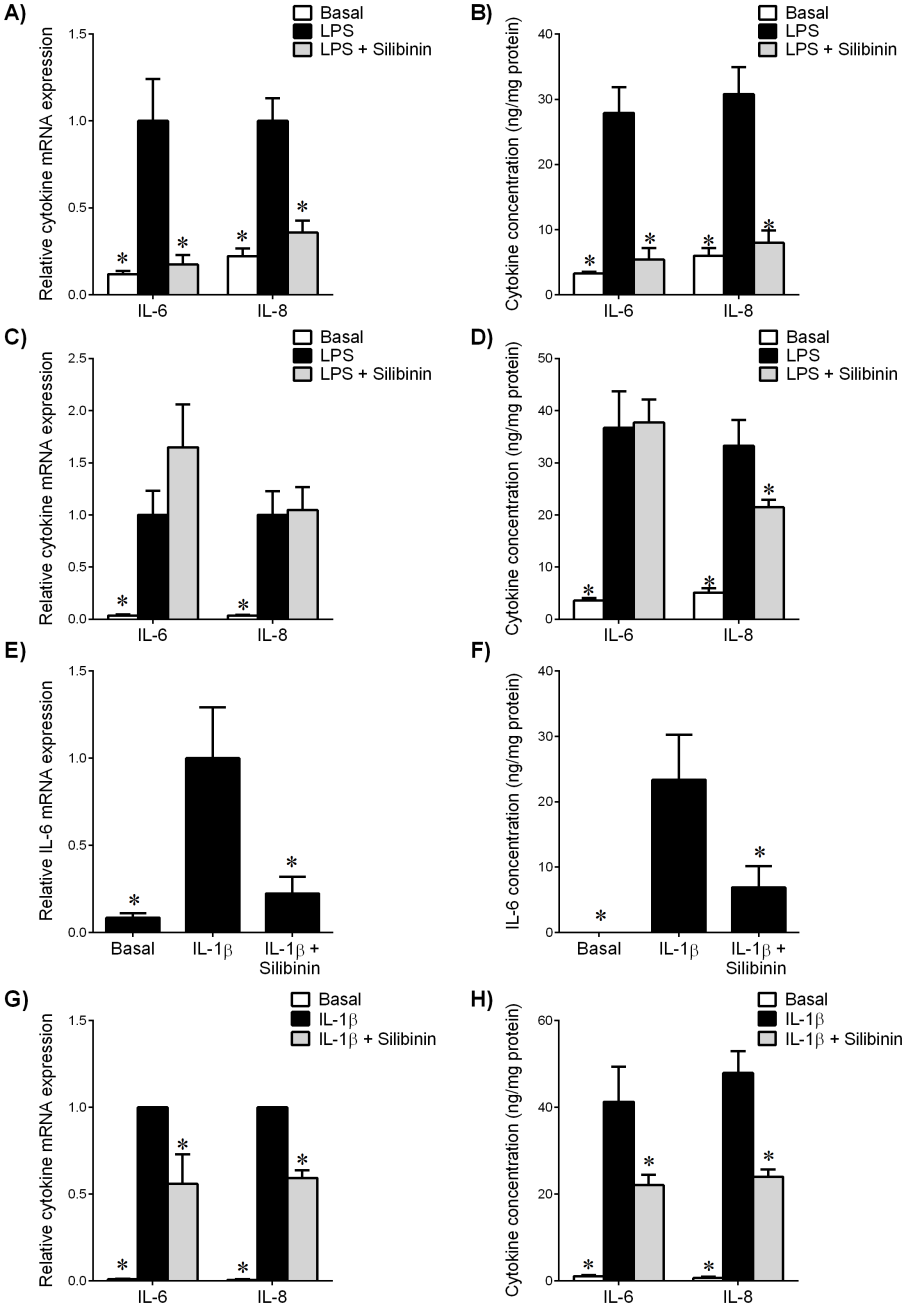
Effect of silibinin on pro-inflammatory cytokines in human gestational tissues. (**A,B**) Fetal membranes and (**C,D**) myometrium were incubated 24 h in the absence or presence of 10 μg/ml LPS with or without 200 μM silibinin (n = 6 patients). (**E,F**) Human amnion cells and (G,H) myometrial cells were incubated 24 h in the absence or presence of IL-1β with or without silibinin (n = 6 patients). (**A,C,E,G**) Gene expression for IL-6 and IL-8 was analysed by qRT-PCR. Cytokine mRNA expression was normalized to GAPDH mRNA expression and the fold change was calculated relative to LPS or IL-1β-stimulated expression. Data is displayed as mean ± SEM (one-way ANOVA). *P<0.05 vs. LPS or IL-1β-stimulated expression. (**B,D,F,H**) The incubation medium was assayed for concentration of IL-6 and IL-8 release by ELISA. Each bar represents mean concentration ± SEM (one-way ANOVA). *P<0.05 vs. LPS or IL-1β-stimulated release.

The effect of silibinin on cytokine expression and release is shown in Figure 1. In fetal membranes, LPS treatment increased IL-6 and IL-8 mRNA expression (Figure 1A) and release (Figure 1B). Treatment with silibinin significantly decreased LPS-induced IL-6 and IL-8 mRNA expression (Figure 1A) and release (Figure 1B). In myometrium, LPS treatment significantly increased IL-6 and IL-8 mRNA expression (Figure 1C) and release (Figure 1D). However, there was no effect of silibinin treatment on LPS-induced IL-6 and IL-8 mRNA expression (Figure 1C). Silibinin significantly decreased LPS-stimulated IL-8 release (Figure 1D) but there was no change in IL-6 expression.

In primary amnion cells, IL-1β-induced IL-6 mRNA expression (Figure 1E) and release (Figure 1F) was significantly attenuated by treatment with silibinin. In myometrial cells, silibinin significantly diminished IL-1β-induced IL-6 and IL-8 mRNA expression (Figure 1G) and release (Figure 1H).

### Effect of silibinin on the COX-prostaglandin pathway in human gestational tissues

Human fetal membranes, primary amnion cells, myometrium, and primary myometrial cells were treated with silibinin as described above. To determine if the effect of silibinin on prostaglandin release occurs at the transcriptional level, the mRNA expression of COX-2, the time-limiting enzyme involved in prostaglandin formation, was measured using qRT-PCR.

**Figure 2:**
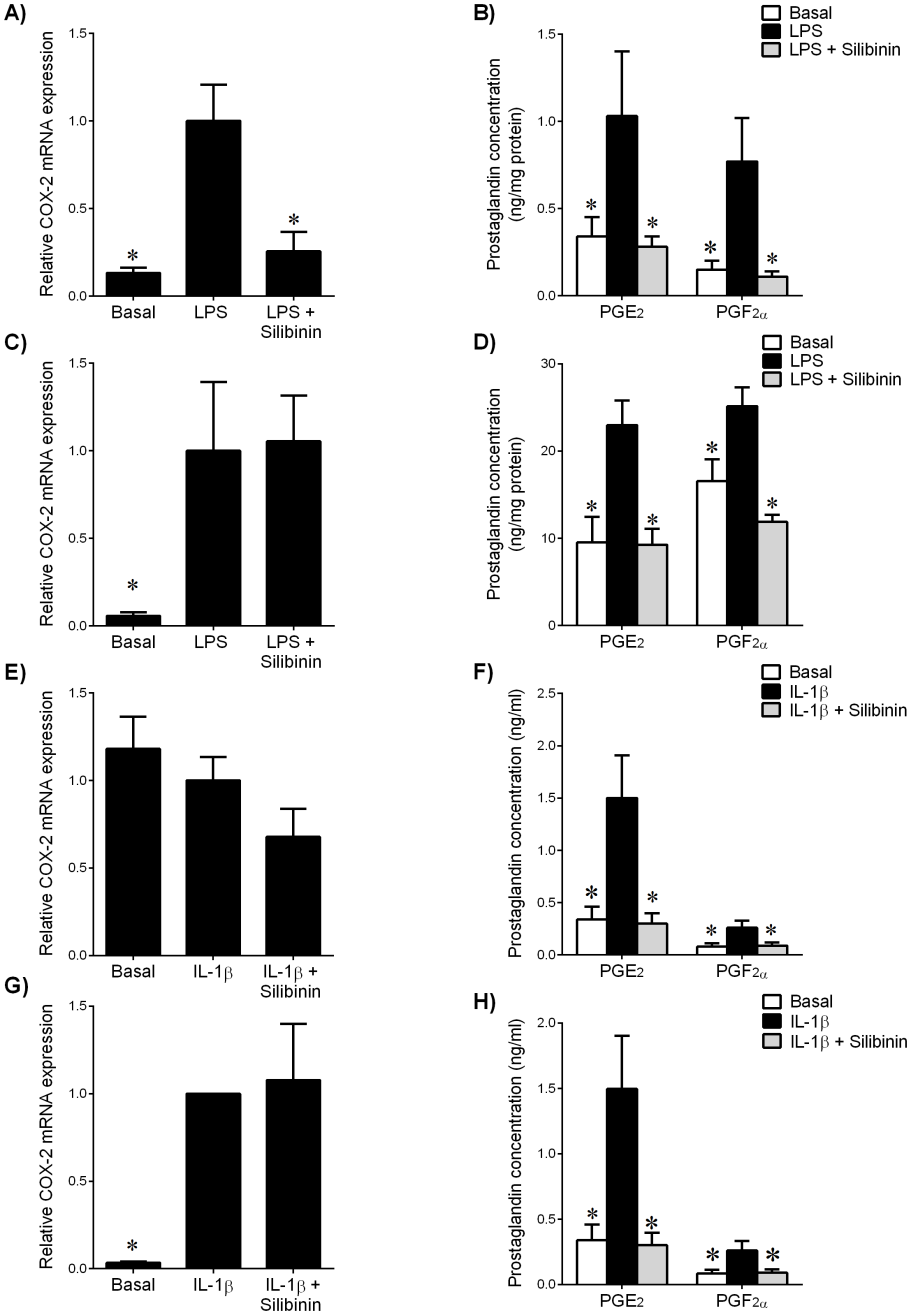
Effect of silibinin on COX-prostaglandin pathway in human gestational tissues. (**A,B**) Fetal membranes and (**C,D**) myometrium were incubated 24 h in the absence or presence of 10 μg/ml LPS with or without 200 μM silibinin (n = 6 patients). (**E,F**) Human amnion cells and (**G,H**) myometrial cells were incubated 24 h in the absence or presence of IL-1β with or without silibinin (n = 6 patients). (**A,C,E,G**) Gene expression for COX-2 was analysed by qRT-PCR. COX-2 mRNA expression was normalized to GAPDH mRNA expression and the fold change was calculated relative to LPS or IL-1β-stimulated expression. Data is displayed as mean ± SEM (one-way ANOVA). *P<0.05 vs. LPS or IL-1β-stimulated expression. (**B,D,F,H**) The incubation medium was assayed for concentration of PGE_2_ and PGF_2α_ release by ELISA. Each bar represents mean concentration ± SEM (one-way ANOVA). *P<0.05 vs. LPS or IL-1β-stimulated release.

In fetal membranes, treatment with LPS increased COX-2 mRNA expression, while silibinin decreased LPS-stimulated COX-2 mRNA expression (Figure 2A). Release of PGE_2_ and PGF_2α_ was increased in the presence of LPS, an effect abrogated with silibinin (Figure 2B). In myometrium, treatment with LPS significantly augmented COX-2 mRNA expression (Figure 2C) and prostaglandin release (Figure 2D). While there was no effect of silibinin on LPS-stimulated COX-2 mRNA expression, silibinin significantly decreased LPS-induced prostaglandin release (Figure 2D).

In primary amnion cells, IL-1β did not induce COX-2 gene expression (Figure 2E); however, IL-1β induced prostaglandin release (Figure 2F), an effect abrogated by silibinin. In primary myometrial cells, there was no change in IL-1β-induced COX-2 mRNA expression (Figure 2G), however silibinin significantly decreased IL-1β-induced PGE_2_ and PGF_2α_ secretion (Figure 2H).

### Effect of silibinin on MMP-9 expression and activity in human gestational tissues

Human myometrium, primary amnion cells, and primary myometrial cells were used to determine the effect of silibinin on MMP-9 expression and activity. Fetal membranes are not responsive to LPS treatment with respect to MMP-9 expression and activity (data not shown). For these studies, MMP-9 gene expression was assessed by qRT-PCR and secreted MMP-9 activity by gelatin zymography.

**Figure 3:**
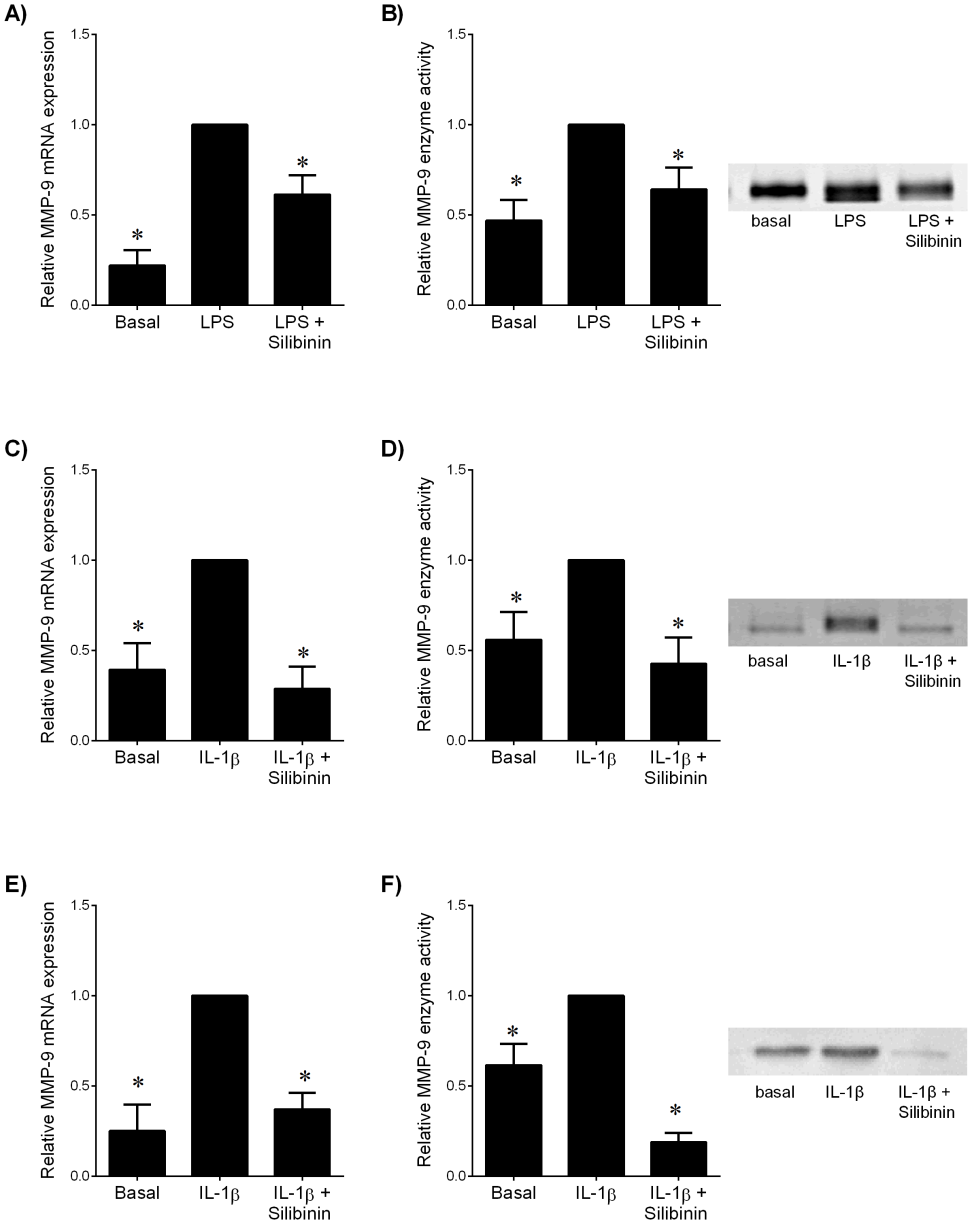
Effect of silibinin on MMP-9 expression in human gestational tissues. (**A,B**) Myometrium was incubated 24 h in the absence or presence of 10 μg/ml LPS with and without 200 μM silibinin (n = 6 patients). (**C,D**) Human amnion cells and (**E,F**) myometrial cells were incubated 24 h in the absence or presence of IL-1β with and without silibinin (n = 6 patients). (**A,C,E**) Gene expression for MMP-9 was analysed by qRT-PCR. MMP-9 mRNA expression was normalized to GAPDH mRNA expression and the fold change was calculated relative to LPS or IL-1β-stimulated expression. Data is displayed as mean ± SEM (one-way ANOVA). *P<0.05 vs. LPS or IL-1β-stimulated expression. (**B,D,F**) The incubation medium was assayed for MMP-9 activity by gelatin zymography. Zymography from one patient per tissue type is shown depicting MMP-9 activity.

In myometrial tissue, LPS treatment significantly increased MMP-9 mRNA expression (Figure 3A) and activity (Figure 3B). Silibinin significantly decreased LPS-induced MMP-9 mRNA expression (Figure 3A) and activity (Figure 3B).

In amnion cells, treatment with IL-1β significantly increased MMP-9 mRNA expression (Figure 3C). Treatment with silibinin significantly decreased IL-1β-induced MMP-9 mRNA expression. Silibinin also decreased IL-1β-induced MMP-9 activity (Figure 3D). Myometrial cells treated with IL-1β showed an increase in MMP-9 mRNA expression (Figure 3E) and activity (Figure 3F). Treatment with silibinin decreased IL-1β-induced MMP-9 mRNA expression (Figure 3E) and activity (Figure 3F).

### Effect of silibinin on fetal membranes from preterm deliveries

**Figure 4:**
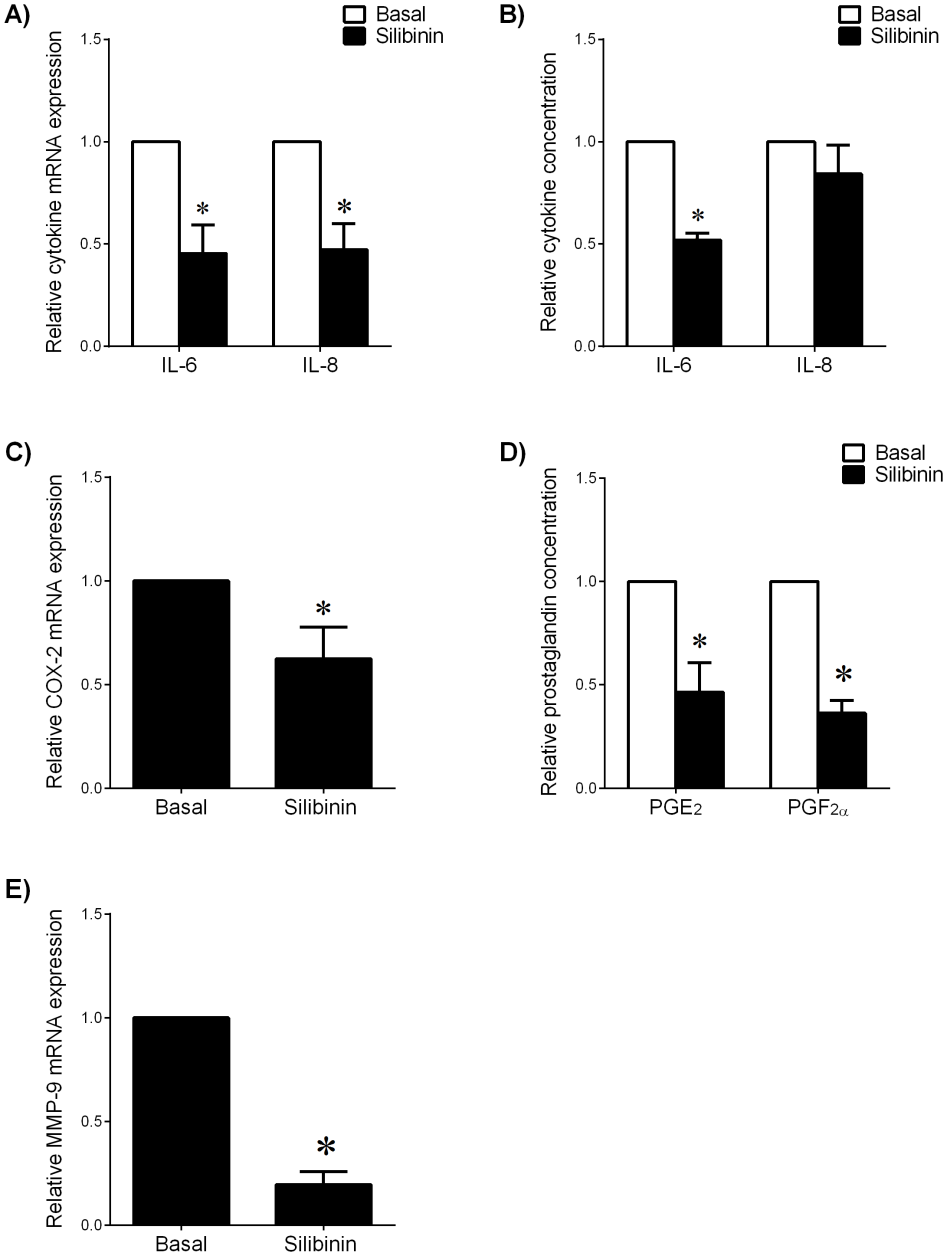
Effect of silibinin on pro-labour mediators in preterm fetal membranes. Preterm fetal membranes with histological chorioamnionitis and following spontaneous preterm labour were incubated for 24 μM silibinin (n = 7 patients). (**A,C,E**) Gene expression for IL-6, IL-8, COX-2 and MMP-9 were analysed by qRT-PCR. mRNA expression was normalized to GAPDH mRNA expression and the fold change was calculated relative to basal expression. Data is displayed as mean ± SEM (one-way ANOVA). *P<0.05 vs. basal expression. (**B,D**) The incubation medium was assayed for concentration of IL-6, IL-8, PGE_2_ and PGF_2α_ release by ELISA. Data was normalised to untreated (basal) levels, which was set at 1. Each bar represents mean ± SEM (one-way ANOVA). *P<0.05 vs. basal release.

The above studies demonstrate that silibinin can decrease LPS- and IL-1β-induced pro-inflammatory mediators *in vitro*. The next aim was to determine if silibinin could decrease pro-labour mediators in an *ex vivo* model using fetal membranes from spontaneous preterm deliveries with and without chorioamnionitis. Cases of chorioamnionitis ranged from mild to severe. Fetal membranes were treated with or without silibinin or vehicle control. The effect of silibinin was found to be equally effective in both non-infected and infected membranes, and thus all subsequent data are combined. The effect of silibinin on cytokine expression and release in preterm fetal membranes is shown in Figures 4A-B. Treatment with silibinin significantly decreased IL-6 and IL-8 mRNA expression (Figure 4A) compared to untreated membranes. Silibinin decreased release of IL-6 (Figure 4B); however the decrease in IL-8 release did not reach significance. Silibinin treatment decreased COX-2 mRNA expression (Figure 4C) and release of prostaglandins (Figure 4D). Silibinin decreased MMP-9 mRNA expression (Figure 4E); however there was no effect on MMP-9 activity (data not shown).

### Effect of silibinin on infection-induced inflammation *in vivo*

The final aim of this study was to determine the efficacy of silibinin in a pregnant mouse model of infection-induced inflammation. Mice were injected with either saline (control), LPS, or LPS with silibinin. After 6 h, placenta and myometrium were collected and pro-inflammatory markers assessed by qRT-PCR. Fetal brains were collected and also assessed for markers of inflammation and fetal brain injury.

**Figure 5:**
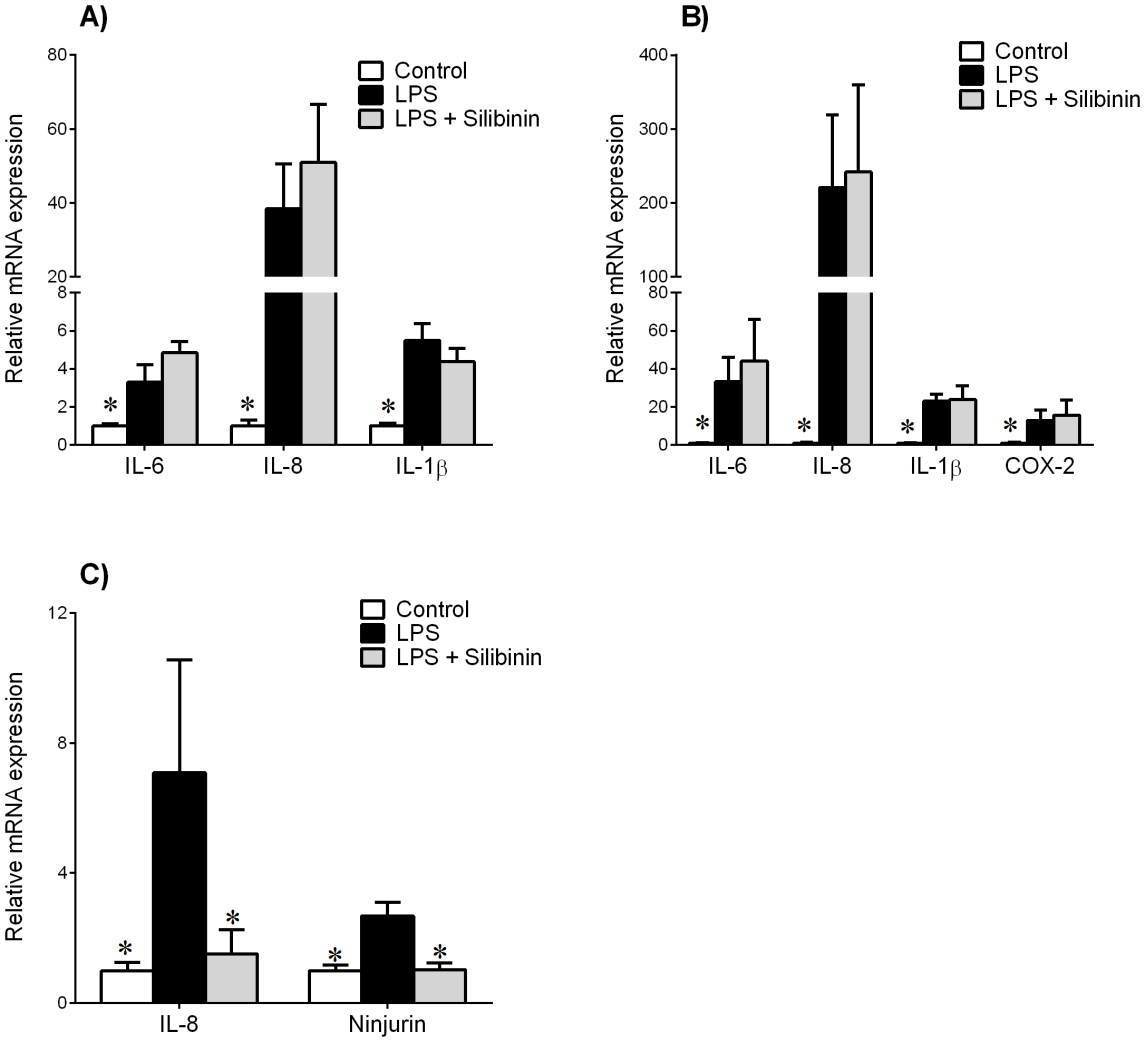
Effect of silibinin in a mouse model of infection-induced preterm birth. Time mated C57BL/6 mice were intraperitoneally injected with either saline (control, n = 7), 50 μg LPS (n = 6), or LPS with 70 mg/kg silibinin (n = 5). Tissues were collected after 6 h. Gene expression for IL-6, IL-8, IL-1β and COX-2 were analysed by qRT-PCR in (**A**) placenta and (**B**) myometrium. Gene expression of IL-8 and ninjurin were analysed in (**C**) fetal brain. Gene expression was normalized to GAPDH mRNA expression and the fold change was calculated relative to control expression. Data is displayed as mean ± SEM (one-way ANOVA). *P<0.05 vs. control gene expression.

In mouse placenta, LPS treatment significantly increased IL-6, IL-8 and IL-1β mRNA expression compared to control (Figure 5A). Treatment with silibinin did not change LPS-stimulated response to these genes. In mouse myometrium, LPS treatment significantly increased IL-6, IL-8, IL-1β and COX-2 mRNA expression (Figure 5B). However, there was no effect of silibinin on LPS-stimulated response in myometrium.

In fetal brain, LPS treatment significantly increased IL-8 and ninjurin mRNA expression compared to control (Figure 5C). Treatment with silibinin significantly decreased LPS-induced IL-8 and ninjurin mRNA expression. We had also assessed other markers of inflammation (IL-6 and IL-1β) and injury (microtubule-associated protein 2 (MAP2), glial fibrillary acidic protein (GFAP) and myelin basic protein (MBP)). However, for these five markers, there was no effect of LPS treatment in the fetal brain compared to controls (data not shown), thus the effect of silibinin was only assessed for IL-8 and ninjurin.

## Discussion

A diet consisting of many serves of fruits and vegetables has been attributed to many health benefits, such as decreased risk of coronary heart disease [63] and decreased incidence of preeclampsia [64], [65]. It is unclear as to why they are beneficial, and it may be due to the presence of dietary phytophenols. In preeclamptic women, the polyphenolic plant flavanoid silibinin has been shown to reduce oxidative metabolism and cytokine production in peripheral blood mononuclear cells [34], [35]. However, the effect of silibinin on mediators of labour in human gestational tissues has not previously been explored. The data presented in this study demonstrate that in human gestational tissues, silibinin decreases LPS and IL-1β-induced mRNA expression and secretion of pro-inflammatory cytokines, COX-2 mRNA expression and resultant prostaglandin release (fetal membranes, but not myometrium), and MMP-9 mRNA expression and activity. In fetal membranes from spontaneous preterm labour and with active infection (confirmed by pathology), silibinin treatment decreased pro-inflammatory cytokine expression and release, COX-2 gene expression and prostaglandin release, and MMP-9 gene expression. We also investigated the ability of silibinin to decrease inflammation and brain injury using a mouse model of infection-induced preterm birth. Fetal brains from C57BL/6 time mated mice treated with silibinin showed a decrease in LPS-induced IL-8 and ninjurin expression. However, silibinin did not change LPS-induced inflammation in placenta or myometrium.

As an inflammatory event, human labour is associated with an induction of pro-inflammatory cytokines such as IL-6, IL-8 and TNF-α, but more so in preterm deliveries [66]. In cases of infection-induced preterm labour, bacterial invasion of the choriodecidual space activates the fetal membranes and decidua to produce pro-inflammatory cytokines; these cytokines stimulate prostaglandin synthesis and release, which also leads to the synthesis and release of MMPs (reviewed in [67]). Prostaglandins are critical in the onset of preterm and term labour, promoting myometrial contractility [13] and cervical ripening [68]. One of the main enzymes responsible for prostaglandin synthesis is COX-2, which is present in human gestational tissues before the onset of labour [13]. COX-2 is induced in tissues after exposure to bacteria as well as inflammatory cytokines, with IL-1β increasing COX-2 expression in myometrium and subsequent prostaglandin production [69]. The activation of pro-inflammatory cytokines, prostaglandins and MMPs that attack the fetal membranes lead to uterine contractions and rupture of membranes. Our study demonstrates that in fetal membranes and primary amnion cells, treatment with silibinin decreased LPS- and IL-1β-induced IL-6 and IL-8 expression, COX-2 expression and subsequent prostaglandin release, and MMP-9 gene expression and activity. In myometrium, silibinin did not affect LPS-induced cytokine mRNA expression, but decreased IL-1β-stimulated cytokine mRNA and release, perhaps suggesting that in myometrium, silibinin may exert anti-inflammatory effects based on the inflammatory stimulus. Silibinin decreased inflammation-induced prostaglandin release but did not affect COX-2 mRNA expression in myometrium; this could be due to silibinin exerting its effects on COX-2 protein expression or activity, which was not assessed in this study. It is also possible that in myometrium silibinin acts by blocking enzymes downstream of COX-2, such as PGH_2,_ synthase or PGE and PGF synthases.

Of all preterm births, spontaneous preterm labour, with intact membranes, accounts for 40–45% of cases [2] and pre-labour rupture of membranes (PROM) occurs in 30–40% of preterm deliveries [70]. While the precise causes are unknown, both are strongly associated with infection and/or inflammation. Women with PPROM commonly begin labour spontaneously; however a proportion remain undelivered for weeks or months, increasing the risk of infection. Abnormal degradation of the fetal membranes has also been proposed as the final pathway of PPROM, with increased MMP-9 activity linked to the increase of inflammation-related cytokines [71]. We determined the effect of silibinin in fetal membranes from preterm deliveries with and without histological infection, with most cases in our study group having PPROM. The one case that did undergo spontaneous preterm labour had acute chorioamnionitis. Silibinin treatment in the preterm fetal membranes decreased pro-inflammatory cytokine expression, COX-2 expression and subsequent prostaglandin release, and MMP-9 gene expression. These results indicate that silibinin can reduce the inflammation already present in the preterm fetal membranes and has some potential as a therapy for threatened cases. However, it may also signify a need for treatment, or a dietary intake that contains silibinin, before PPROM and preterm labour occurs. Myhre *et al* report that intake of garlic is associated with decreased risk of preterm delivery, and dried fruits, particularly raisins, is associated with reduced risk of PPROM [72]. The effects of garlic were attributable to the component allicin, which has strong antimicrobial properties and may have lowered the overall inflammation level.

In survivors of preterm birth, perinatal brain damage is a primary cause of developmental delay and lifelong neurological impairments such as mental retardation and cerebral palsy [6], [7]. Antenatal infection has been linked to white matter damage and cerebral palsy; in babies born within one hour of membrane rupture (with low risk of infection subsequent to the rupture) whose placentas had histologic chorioamnionitis, the presence of leukocytes in the umbilical cord and chorionic plate were associated with an eleven-fold increase in the risk of white matter damage [73]. Studies involving mice show that inflammation-induced preterm birth caused neuropathology in the fetal brains, but not in preterm mice induced by a non-inflammatory method, indicating that it is not the process of preterm birth that affects neuronal morphology [74]. In this study, gestational day 15.5 mice were given an intraperitoneal injection of LPS with or without silibinin and compared to a basal group. In the fetal brain, a marker of inflammation (IL-8) and a marker of neuronal damage (ninjurin) were assessed. Silibinin significantly decreased LPS-induced mRNA expression of ninjurin and IL-8 in the fetal brain. Ninjurin was used as an indicator of neuropathy and inflammation in the fetal brain. It is associated with the increased activity of microglial cells, the immune cells of the brain, during inflammation and tissue remodelling [75]. It also has been found to regulate the migration of macrophages to sites of inflammation in the central nervous system [76]. The phytonutrient supplement study to prevent preeclampsia [77] demonstrated a non-significant reduction in neonatal death, NICU admission and respiratory distress syndrome with the phytonutrient group, promising improved neonatal outcome if a large cohort could be achieved. The ability to reduce the expression of IL-8 and ninjurin in the fetal brain demonstrates that silibinin has great potential as a treatment for infection-induced preterm birth.

Our study demonstrated that *in vitro*, silibinin decreases LPS and IL-1β-induced inflammation in human gestational tissues, and *in vivo*, decreased inflammation and injury in the fetal brain; however, in the mouse, there was no effect of silibinin on LPS-stimulated inflammation in placenta and myometrium. All tissues were collected 6 h after injection of treatments; it is known that a low dose of LPS in a mouse model causes fetal brain injury while not inducing preterm birth [78] (intrauterine infusion of 50 μg LPS rather than high dose of 250 μg). A number of issues could be addressed in future studies: can silibinin delay infection-induced preterm birth, and decrease inflammation at the later time point; would a higher dose of silibinin achieve the reduction of inflammation in maternal tissues; what is the effect of silibinin in the pregnant mouse without LPS; and should silibinin be given as a treatment at the start of pregnancy, regularly, before onset of infection. It is this last point that would be of most interest in human trials to determine whether silibinin, if taken regularly as a supplement or part of a diet, could be a treatment for the prevention of infection-induced preterm birth.

While we must be appropriately cautious to suggest administering silibinin to pregnant women during critical periods of fetal development, pilot studies have previously been performed in humans as a colorectal cancer chemopreventative agent. Initially, studies were performed in mice where dietary feeding of silibinin at 75 or 150 mg/kg for 60 days after receiving a tumour xenograph implant, or fed 3 weeks before tumour implantation and 63 days after, showed that silibinin decreased tumour volume, and that animals did not show weight loss or reduced food consumption [79]. This led to the pilot study by Hoh *et al*, confirming that oral administration of silibinin (silibinin formulated with phosphatidylcholine, as silipide capsules) in humans at daily doses (extrapolated by the mice studies) of up to 1.44 mg for a week, is safe [80], and achieved levels in the colorectal tract similar to those known to exert pharmacologic activity. Of note, our study involved a single dose of silibinin at 70 mg/kg at the same time as LPS injection; repeat doses of silibinin, perhaps orally, to the pregnant mouse before infection-induced preterm birth could possibly give the desired effect of reducing the inflammation associated with preterm birth. While evidence from the previous studies bodes well for safe consumption, further studies are required to determine any side effects and the ability of silibinin to cross the placenta and act on the fetus. For example, silibinin has known anti-tumoral effects mediated, among others, by inhibiting cell cycle progression [81]. Therefore, although the data in our study is promising, we must be cautious before recommending a therapeutic regime using silibinin or other flavonoids in pregnancy, due to the potential existence of serious side-effects on the fetus.

The transcription factor nuclear factor-kappa B (NF-κB) is a common, central pathway involved in promoting the formation of pro-inflammatory and pro-labour mediators in human gestational tissues [82], [83]. For example, we have previously shown that inhibition of NF-κB activity in ex situ human gestational tissues, suppresses the formation of labour-mediating effectors, including pro-inflammatory cytokines, COX-prostaglandins and ECM enzymes. Thus, it is possible that silibinin is exerting its anti-inflammatory actions by inhibiting the activity of NF-κB. Indeed, in non-gestational tissues, silibinin has been shown to exert its effects by inhibiting the activity of NF-κB [27], [40], [55].

In conclusion, we demonstrate that silibinin can reduce infection and inflammation-induced pro-labour mediators in human fetal membranes and myometrium, and can reduce pro-inflammatory cytokines, prostaglandins and gene expression of MMP-9 in preterm fetal membranes both with active infection and from spontaneous preterm labour. Of most promise is our *in vivo* data, where silibinin decreased inflammation and injury in fetal brains in an infection-induced model of inflammation in the pregnant mouse. While the *in vivo* data is preliminary, the ramifications of our findings is that silibinin could be a novel treatment that not only reduces the inflammation associated with preterm labour, but also improves the adverse outcomes that are commonly linked with this global predicament. However, further studies are required to fully understanding the mechanisms of action of silibinin and importantly its effects on the fetus.
